# AURKA Increase the Chemosensitivity of Colon Cancer Cells to Oxaliplatin by Inhibiting the TP53-Mediated DNA Damage Response Genes

**DOI:** 10.1155/2020/8916729

**Published:** 2020-08-10

**Authors:** Baocong Shan, Ran Zhao, Jian Zhou, Minghui Zhang, Xiaoyu Qi, Tianzhen Wang, Jinan Gong, Yiqi Wu, Yuanyuan Zhu, Weiwei Yang, Yang Zhang, Guangyou Wang, Xiaobo Li

**Affiliations:** ^1^Department of Pathology, Harbin Medical University, Harbin 150081, China; ^2^Department of Oncology, Chifeng City Hospital, Chifeng 024000, China; ^3^Hospital of Stomatology, Zunyi Medical University, Zunyi 563099, China; ^4^Department of Pathology, Shenyang Fourth People's Hospital, Shenyang 110031, China; ^5^Department of Neurobiology, Harbin Medical University, Harbin 150081, China

## Abstract

AURKA, a cell cycle-regulated kinase, is associated with malignant transformation and progression in many cancer types. We analyzed the expression change of AURKA in pan-cancer and its effect on the prognosis of cancer patients using the TCGA dataset. We revealed that AURKA was extensively elevated and predicted a poor prognosis in most of the detected cancer types, with an exception in colon cancer. AURKA was elevated in colon cancer, but the upregulation of AURKA indicated better outcomes of colon cancer patients. Then we revealed that undermethylation of the AURKA gene and several transcription factors contributed to the upregulation of AURKA in colon cancer. Moreover, we demonstrated that AURKA overexpression promoted the death of colon cancer cells induced by Oxaliplatin, whereas knockdown of AURKA significantly weakened the chemosensitivity of colon cancer cells to Oxaliplatin. Mechanistically, AURKA inhibited DNA damage response by suppressing the expression of various DNA damage repair genes in a TP53-dependent manner, which can partly explain that ARUKA is associated with a beneficial outcome of colon cancer. This study provided a possibility to use AURKA as a biomarker to predict the chemosensitivity of colon cancer to platinum in the clinic.

## 1. Introduction

Aurora Kinase A (AURKA) is a cell cycle-regulated kinase involved in centrosome maturation, mitotic entry, bipolar spindle assembly, and chromosome separation [1]. The elevated expression of AURKA is frequently reported in many cancer types [2]. AURKA, alone or combined with other factors, can trigger cell malignant transformation [3, 4] and promote the malignant phenotype of cancer cells [5, 6]. AURKA shows oncogenic activity by regulating multiple oncogenic and tumor-suppressive proteins [7]. Of these proteins, tumor suppressor TP53 has been intensively studied. Phosphorylation of TP53 at Ser215 and Ser315 by AURKA results in TP53 degradation by MDM2-mediated ubiquitination and abrogation of TP53 DNA binding/transactivation activity, respectively [8, 9]. In turn, TP53 downregulation increases the expression of ARUKA at both transcriptional and posttranslational levels [10, 11]. Negative feedback regulation between AURKA and TP53 greatly promotes carcinogenesis and progression.

Maintaining genome stability by transactivating the DNA damage response (DDR) genes is the critical mediator of TP53-dependent tumor suppression [12, 13]; thus, TP53 deficiency causes the loss of various DDR mechanisms and thereby facilitates genome instability and cancer development [13]. Meanwhile, platinum-induced DNA damage can trigger the DDR, which is a major contributor to chemoresistance to platinum [14]. In view of the association between AURKA, TP53, and DDR, the upregulated AURKA in cancer might promote the cancer progression but meanwhile enhance the chemosensitivity of DNA damage-inducing drugs in the clinic.

In this study, we analyzed the expression state and regulation mechanism of AURKA in colon cancer. We also tested the effect of dysregulated AURKA on chemosensitivity to the platinum drug and explored the underlying molecular mechanism in colon cancer. These results provided a novel insight into the function of AURKA in cancer.

## 2. Material and Methods

### 2.1. Dataset and Processing

The data of AURKA mRNA expression in 18 types of cancers and matched normal tissues were downloaded from The Cancer Genome Atlas (TCGA) database, including Bladder Urothelial Carcinoma (BLCA), Breast Invasive Carcinoma (BRCA), Cervical Squamous Cell Carcinoma and Endocervical Adenocarcinoma (CESC), Colon Adenocarcinoma (COAD), Head and Neck Squamous Cell Carcinoma (HNSC), Kidney Renal Clear Cell Carcinoma (KIRC), Kidney Renal Papillary Cell Carcinoma (KIRP), Liver Hepatocellular Carcinoma (LIHC), Lung Adenocarcinoma (LUAD), Lung Squamous Cell Carcinoma (LUSC), Pancreatic Adenocarcinoma (PAAD), Prostate Adenocarcinoma (PRAD), Rectum Adenocarcinoma (READ), Sarcoma (SARC), Skin Cutaneous Melanoma (SKCM), Stomach Adenocarcinoma (STAD), Thyroid Carcinoma (THCA), and Uterine Corpus Endometrial Carcinoma (UCEC). The correlation between the AURKA level and the overall survival (OS) of cancer patients was also analyzed through the GEPIA (http://gepia.cancer-pku.cn/index.html). The mRNA expression data are shown in Supplementary 2. We compared the expression level of AURKA mRNA by calculating the mean value and standard deviation. The effect of AURKA copy number variant (CNV) on AURKA expression level was also analyzed based on the Colon Adenocarcinoma (COAD) data from the TCGA database. The effect of methylation on the expression of AURKA was assessed using the MEXPRESS data (https://mexpress.be/). The transcription factor- (TF-) targeting ARUKA was screened based on the Chip-Seq data in the UCSC databank (http://genome.ucsc.edu/ENCODE/). The targeted regulatory capacity of TP53 on DDR genes was assessed using the Cistrome Data Browser (http://cistrome.org/db/#/). Meanwhile, the correlation between AURKA and TF genes was analyzed in colon cancer through the GEPIA (http://gepia.cancer-pku.cn/index.html).

### 2.2. Cell Culture

Two colon cancer cell lines (SW1116 and HCT116) and 293TN cell line were used in this study. Missense mutation presents in TP53 in SW1116, whereas HCT116 has a wild-type TP53 according to the Cancer Cell Line Encyclopedia (CCLE) (https://portals.broadinstitute.org/ccle/about). Additionally, all the DDR genes involved in our study are the wild-type but for BRCA2, which has a frameshift in HCT116. They were cultured using Dulbecco's Modified Eagle Medium (DMEM; HyClone, Logan, UT, USA) with 10% fetal bovine serum (FBS; Invitrogen, Carlsbad, CA, USA), 100 *μ*g/ml streptomycin, and 100 IU/ml penicillin at 37°C in a humidified atmosphere containing 5% CO_2_. Cells used to detect phosphorylated TP53 were treated with a specific proteasome inhibitor MG132 (Sigma, St. Louis, MO, USA, 10 *μ*M for six hours).

### 2.3. The Construction of Stable Cell Lines

Overexpression or knockdown of AURKA was achieved by using lentivirus particles to infect colon cancer cells described as before [15]. In brief, the ORFs of AURKA cloned by PCR and synthesized shRNA against AURKA were inserted into Plvx-Puro and SHC201 vectors, respectively. The scramble sequences were inserted into these vectors to be used as control. These vectors were transfected into 293TN cells with the packing vectors (System Bioscience, Mountain View, CA, USA) to get pseudo lentiviral particles. After being filtered and concentrated by PEG precipitation (System Bioscience), lentiviral particles were added to the culture medium to infect colon cancer cells for 12 h. After routine culture for 72 h, the stable cells were selected and purified by puromycin (2 *μ*g/ml).

### 2.4. MTT Assay

Colon cancer cells were seeded in 96-well plates at a density of 5000 cells per well and incubated overnight. The culture medium was replaced with fresh culture medium containing a different concentration of Oxaliplatin (0, 20, 40, 80, and 160 *μ*g/ml) with 5 replicates each. After 48 h of incubation, 20 *μ*l MTT (5 g/l) was added to each well for 4 h in the incubator. The supernatant was removed, and 150 *μ*l DMSO was added to each well. After being vibrated for 10 min, the plate was read on a microplate reader at 570 nm to calculate the cell viability rate. All assays were replicated three times. The result was analyzed using the cell viability percentage (the total number of viable cells at each drug concentration relative to the number of viable cells treated with the solvent control).

### 2.5. Western Blot

Total proteins were extracted from colon cancer cells using the RIPA buffer (Beyotime Institute of Biotechnology, Shanghai, China). 10 *μ*g protein was separated in SDS-PAGE gel by electrophoresis and transferred onto PVDF membrane. The blots were blocked by 5% BSA at 4°C overnight. The membrane was incubated with primary antibodies: AURKA rabbit polyclonal antibody (ProteinTech, Wuhan, China. No. 10297-1-AP) diluted at 1 : 1000, TP53 rabbit polyclonal antibody (Proteintech, Wuhan, China. No.10442-1-AP) diluted at 1 : 1000, phospho-TP53 (Ser315) mouse monoclonal antibody (Santa Cruz Biotechnology. No.sc-135772), MDM2 rabbit polyclonal antibody (Proteintech, Wuhan, China. No. 19058-1-AP), and GAPDH mouse monoclonal antibody (ProteinTech, Wuhan, China. No. 60004-1-Ig) diluted at 1 : 5000. After washing, the membranes were incubated with peroxidase-conjugated secondary antibody (Santa Cruz Biotechnology) for 1 h at 37°C. The ECL system (Thermo Scientific, Rockford, IL, USA) was used to visualize the blots. All assays were replicated three times.

### 2.6. Real-Time PCR

Total RNA was extracted from colon cancer cells using TRIzol (Invitrogen, Carlsbad, CA, USA). EasyScript® Reverse Transcriptase (TransGen Biotech Co., Beijing, China) was used to reverse RNA into cDNA. The level of DDR gene (ATR, XLF, XRCC1, RPA1, BRCA2, and RAD51) was quantified using the SYBR Green PCR mix (Bioresearcher, Beijing, China) through CFX96TM Real-Time System (Bio-Rad). The reaction mixture underwent 38 cycles consisting of denaturation for 10 s at 95°C and annealing and prolongation for 30 s each at 60°C. GAPDH was used as the endogenous controls. All assays were replicated three times. The primers used for PCR are shown in Supplementary 3.

### 2.7. Statistics Analysis

The expression of AURKA in a different type of tumors and the differential expression of genes between two groups were analyzed by a two-sided Student's *t*-test. Survival analyses were conducted with the Kaplan-Meier method using the log-rank test, and the median value separation model based on the AURKA expression is presented. The hazard ratio was calculated based on the Cox PH model. The correlation between methylation status and AURKA expression was analyzed using the Pearson correlation and Wilcoxon rank-sum test. Pearson's correlation and *Z* test were used to analyze the correlation between AURKA and TFs. The effect of CNV on AURKA expression was assessed by the Kruskal-Wallis test. MTT results were analyzed using variance analysis (∗*p* < 0.05, ∗∗*p* < 0.01, ∗∗∗*p* < 0.001).

## 3. Results

### 3.1. AURKA Was Upregulated and Predicted a Beneficial Outcome in Colon Cancer

**Figure 1:**
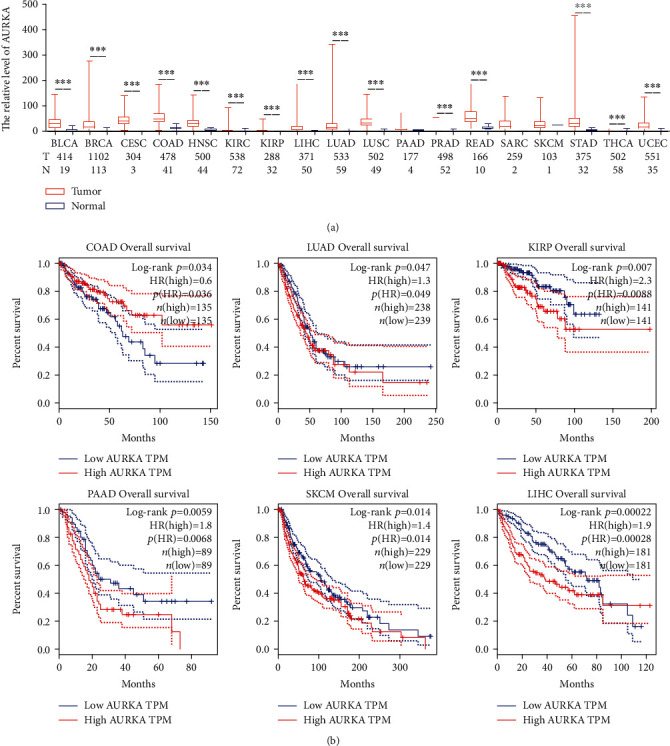
AURKA was upregulated in colon cancer and predicted a benefit outcome. (a) Compared with the matched normal tissues, AURKA was significantly upregulated in cancer tissues in 15 out of 18 cancer types. (b) AURKA expression level was adversely correlated with OS in 5 of 15 cancers but positively correlated with OS in COAD.

To explore the effect of AURKA on cancer progression and prognosis, we firstly employed the TCGA dataset to analyze the mRNA expression of AURKA in 18 types of tumors. Compared with the matched normal tissues, AURKA was significantly upregulated in cancer tissues in 15 out of 18 cancer types (Figure 1(a)). Next, we assessed the correlation between the AURKA level and overall survival (OS) in 15 cancer types using the GEPIA. We showed that the AURKA level was adversely correlated with OS in 5 of 15 cancers, including LUAD, KIRP, PAAD, SKCM, and LIHC. However, a high level of AURKA was associated with a longer OS in COAD (Figure 1(b)). These results suggested that AURKA overexpression might play an important role during the carcinogenesis and progression of cancer; however, the elevated expression of AURKA predicted a beneficial outcome only in colorectal cancer.

### 3.2. DNA Undermethylation and Several Transcription Factors Might Contribute to the Elevated Expression of AURKA in Colon Cancer

**Figure 2:**
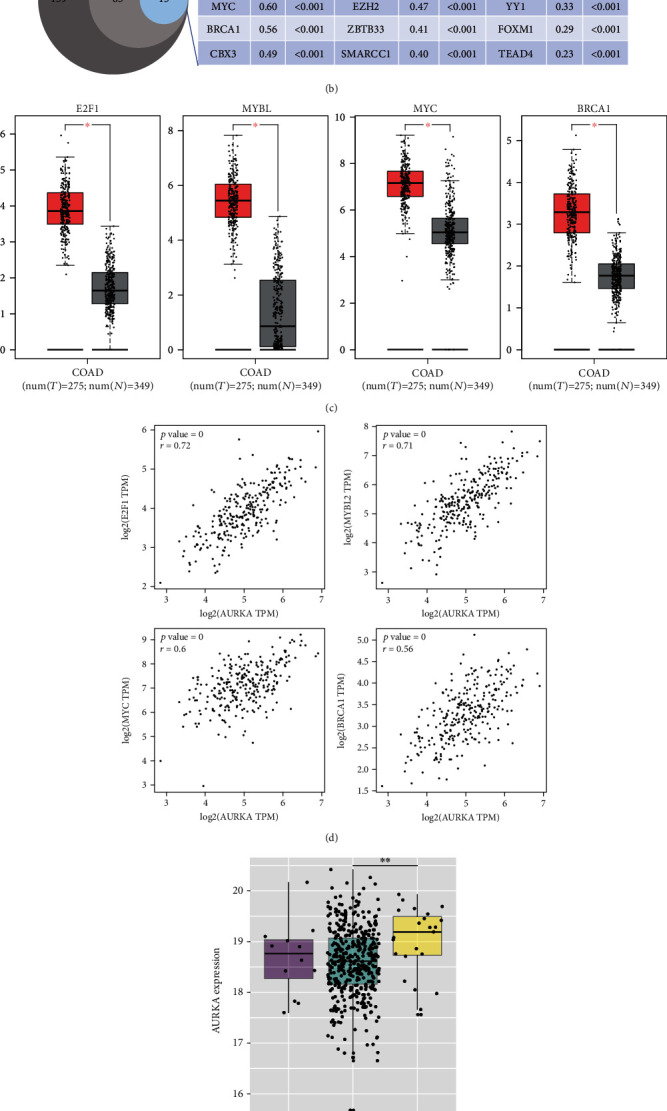
Undermethylation, upregulation of TFs, and gene amplification potentially contributed to the elevated expression of AURKA. (a) Five methylation sites in AURKA DNA were significantly adversely correlated with the level of AURKA. (b) Based on the public data analysis, a total of 159 TFs potentially regulated AURKA transcription. The expression of 85 TFs was positively correlated with the level of AURKA. Moreover, 15 of them have been identified to be overexpressed in colon cancer tissues compared with the matched normal tissues. (c, d) The expression of the top four TFs highly correlated with AURKA was higher in colon cancer tissues compared with normal tissues. (e) The expression level of AURKA in the AURKA CNV gain group was significantly higher than that in the AURKA CNV neutral group in COAD.

To explore the mechanism by which AURKA was upregulated in colon cancer, we firstly analyzed the effect of methylation status on AURKA expression. By using the MEXPRESS, there were 21 methylation sites in the AURKA gene identified. Of them, 5 methylation sites were significantly adverse correlated with the level of AURKA (Figure 2(a)). Meanwhile, we screened the potential TFs activating AURKA expression based on the Chip-Seq data using the UCSC database and found that a total of 159 TFs potentially regulate AURKA transcription. Of them, the expression of 85 TFs was positively correlated with the level of AURKA in colon cancer tissues according to the GEPIA correlation analysis. Moreover, 15 of them have been identified to be overexpressed in colon cancer tissues compared with the matched normal tissues through the GEPIA expression analysis (Figure 2(b)). The top four TFs highly correlated with AURKA (*r* > 0.5, *p* < 0.01) were E2F1, MYBL2, MYC, and BRCA1. The expression and correlation with AURKA of these four TFs are shown in Figures 2(c) and 2(d). We also analyzed the effect of AURKA CNV on the expression level of AURKA. The result indicated that the expression level of AURKA in the AURKA CNV gain group was much higher than that in the AURKA CNV neutral group in COAD, whereas there was no difference between the AURKA CNV loss and CNV neutral group (Figure 2(e)). But the incidence of CNV gain was lower in colon cancer patients. These results indicated that undermethylation, the elevated TFs, and gene amplification might contribute to the elevated expression of AURKA in colon cancer.

### 3.3. AURKA Increased the Chemosensitivity of Colon Cancer Cells to Oxaliplatin

**Figure 3:**
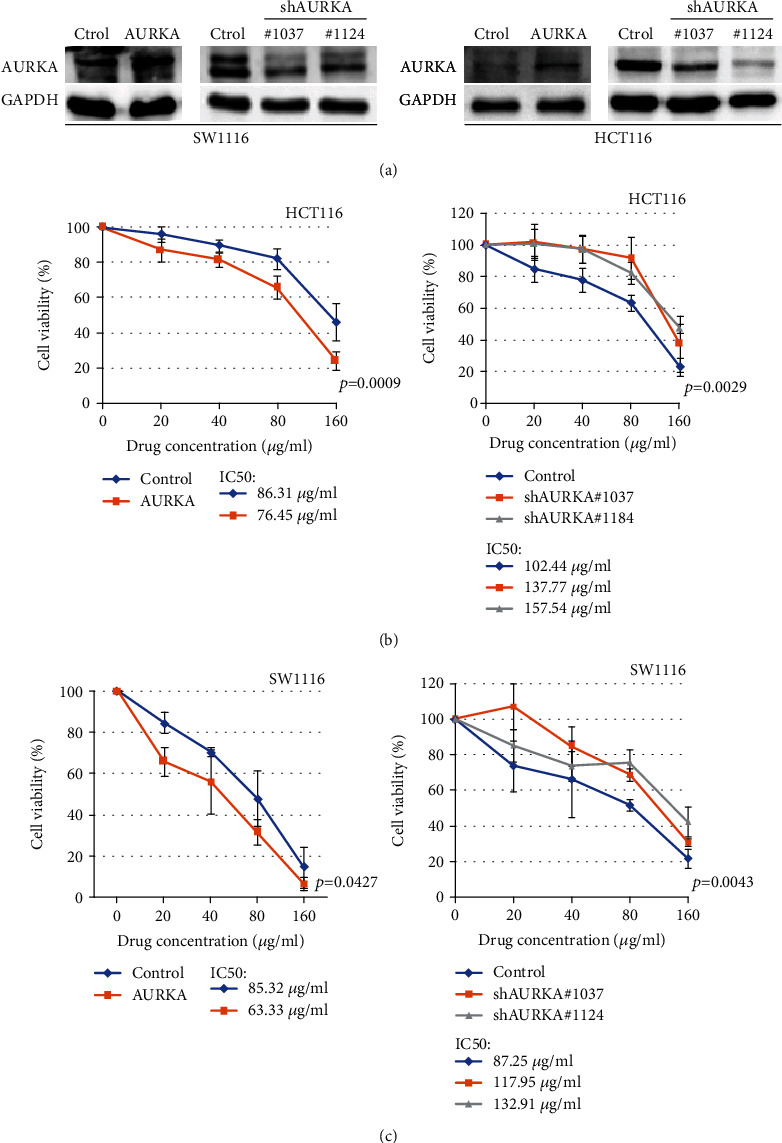
AURKA increased the chemosensitivity of colon cancer cells to Oxaliplatin. (a) AURKA was upregulated or knocked down in two cancer cell lines. (b, c) AURKA overexpression promoted the death of HCT116 and SW1116 colon cancer cells induced by Oxaliplatin, whereas knockdown of AURKA significantly weakened the response of colon cancer cells to Oxaliplatin.

We found that upregulated AURKA was associated with the improved prognosis of colon cancer patients; thus, we speculated that if AURKA increases chemosensitivity of platinum by increasing the genomic instability in colon cancer. We firstly constructed the stable cell lines with AURKA overexpression or knockdown (Figure 3(a)) and then assessed the effect of AURKA on the chemosensitivity of colon cancer cells. The result indicated that AURKA overexpression promoted the death of HCT116 and SW1116 colon cancer cells induced by Oxaliplatin, whereas knockdown of AURKA significantly weakened the response of colon cancer cells to Oxaliplatin (Figures 3(b) and 3(c)). These results showed that AURKA may improve the prognosis of colon cancer patients by increasing the chemosensitivity of colon cancer cells to the DNA-damaging drug.

### 3.4. AURKA Downregulated the Expression of DDR Genes by Inhibiting TP53

**Figure 4:**
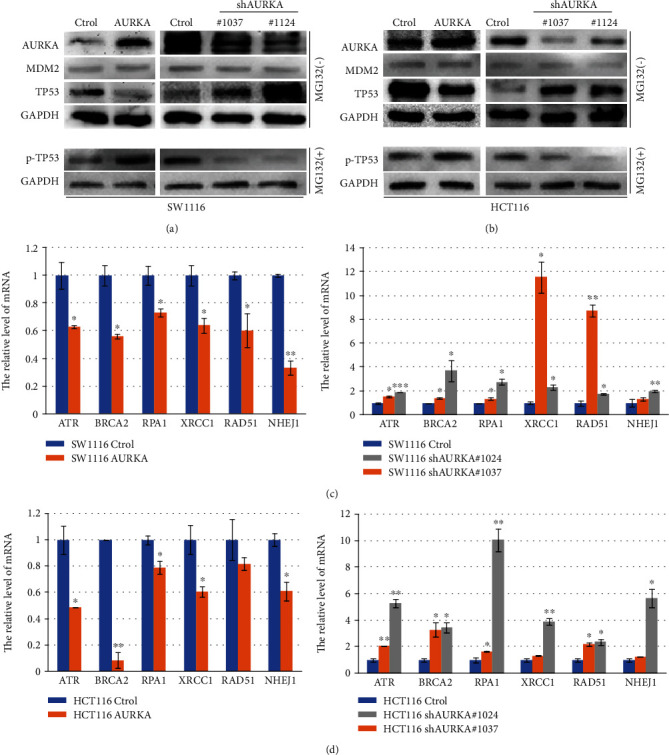
AURKA downregulated the expression of DDR genes by inhibiting TP53. (a, b) Overexpression of AURKA promoted the phosphorylation of TP53 and decreased the level of total TP53, whereas knockdown of AURKA reduced the phosphorylation of TP53 and increased the level of total TP53 in colon cancer cells by immunoblot. AURKA had no effect on the expression of MDM2. (c, d) Six representative DDR genes were downregulated in colon cancer cells with AURKA overexpression but upregulated when knocking down AURKA in colon cancer cells by real-time PCR.

Previous research showed that AURKA inhibits the expression of TP53, which mediates the expression of DDR genes at the transcriptional level. We detected the effect of AURKA on TP53 expression by immunoblot in colon cancer cells. The result indicated that TP53 was downregulated when AURKA was overexpressed, whereas upregulated when AURKA was knocked down in colon cancer cells (Figures 4(a) and 4(b)). Next, we screened a set of DDR genes that play an important role in DNA damage induced by chemotherapeutics. Meanwhile, most of them function after the activation of TP53 [16]. Using the Cistrome Data Browser, we assessed the transcriptional regulatory potential of TP53 on these genes and found some of them had higher scores in two sets of data with high-quality control (Supplementary 4). We then applied real-time PCR to verify the expression of six representative genes, ATR, XLF, XRCC1, RPA1, BRCA2, and RAD51. The results indicated that the six DDR genes were downregulated in colon cancer cells with AURKA overexpression but upregulated when knocking down AURKA in colon cancer cells (Figures 4(c) and 4(d)), which implied that AURKA increased the chemosensitivity of colon cancer cells to DNA damage-inducing drugs by inducing the degradation of TP53 and then decreasing the expression of DDR genes.

## 4. Discussion

In this study, we evaluated the expression of AURKA in 18 types of tumor tissues and matched normal tissues. The result indicated that AURKA was upregulated in most tested cancer types compared with their normal tissues. OS analysis showed that higher AURKA was correlated with a worse outcome of most of the cancer types, whereas it only indicated a favorable outcome in colon cancer. The prognostic role of AURKA has ever been assessed in colorectal cancer patients by a research team in 2014 [17]. Despite the lack of statistical significance, they still put forward that AURKA may have a positive effect on survival and emphasized the necessity to study the effect of AURKA on response to treatment [17]. Further study showed that undermethylation and upregulation of TFs potentially contribute to the elevated expression of AURKA in colon cancer at least partly. Some studies indicated that gene amplification is another contributor to the elevated AURKA [18, 19]. We also identified that gene amplification in colon cancer patients can result in AURKA upregulation; however, its incidence rate was very low in colon cancer patients. Finally, we demonstrated that AURKA might improve the prognosis of colon cancer patients by increasing the chemosensitivity of colon cancer to Oxaliplatin via inhibiting the DDR. Our results uncovered the double-edged sword effects of AURKA by inhibiting TP53 in colon cancer.

The genomic instability has been recognized as a hallmark of cancer, and it is associated with carcinogenesis and progression of cancer [20, 21]. AURKA functions as an oncogene during the development of multiple malignant tumors by inducing centrosome amplification and genomic instability [3, 22]. In colon cancer, the overexpressed AURKA is the contributor to chromosomal instability [23, 24]. Moreover, AURKA has been revealed to impair the function of DNA damage repair through inhibiting the expression of DDR genes, such as RAD51 and BRCA1/2 [25–27]. In addition to the DDR genes involved in Homologous Recombination Repair (HRR), TP53 showed the transcriptional regulatory potential on Mismatch Repair (MMR) genes according to the binding scores from the Chip-Seq data (Supplementary 5). The inhibitory effect of AURKA on TP53, which has been demonstrated to transcriptionally activate many DDR genes, enlarges the potential of AURKA facilitating DNA damage [13]. Some studies also indicate that TP53 is essential for chemoresistance rendered by AURKA [28, 29]. In order to verify the function of TP53 during this process, we, respectively, assessed the correlation between AURKA level and OS in patients with wild-type or mutant TP53. The results indicated that the patients with the higher AURKA had a longer OS time in TP53 wild-type groups, although only a marginal significance was achieved due to the reduced number of samples. But no difference was found in TP53 mutant groups (Supplementary 1).

The current research supports that AURKA is involved in colon carcinogenesis through promoting genomic instability, but the increased AURKA provides a good chance for enhancing the sensitivity of chemotherapy based on DNA damage-inducing drugs. The effect of AURKA on chemosensitivity has been studied in different cancer types. Up to now, they all concluded that AURKA impaired the chemosensitivity, which is the exact opposite of our finding. For example, it was reported that inhibiting AURKA enhances the chemosensitivity of cancer cells to the taxane and paclitaxel [30, 31], cisplatin [32], doxorubicin [33, 34], and 5-fluorouracil (5-Fu) [35]. In particular, platinum chemosensitivity is inhibited by AURKA in various cancers including ovarian cancer [36], hepatocellularcarcinoma [37], medulloblastoma [38], acute myeloid leukemia [39]as well as head and neck cancer [40]. Our finding that AURKA increased the platinum chemosensitivity in colon cancer was different from the previous studies in other cancer types, which coincided with our finding that higher AURKA indicated better prognosis only in colon cancer but not in other cancers. Though cancer stem cell is a small subpopulation of cancer cells, AURKA silencing sensitized the response of colorectal cancer stem cell (CR-CSC) to Oxaliplatin by upregulating antiapoptotic factors [29], which is different from our findings in colon cancer cells. The difference might be associated with heterogeneity induced by tumor microenvironment and genomic instability [41]. AURKA-mediated TP53 inhibition might result in different consequence in different genetic contexts. However, this hypothesis might be determined by further experiments.

## 5. Conclusion

AURKA was upregulated in various cancer types but only positively correlated with the prognosis of colon cancer patients. The mechanism might be that AURKA improves the chemosensitivity of colon cancer cells to Oxaliplatin by inhibiting the expression of TP53-regulated DDR genes and then facilitating DNA damage. This study provides a possibility to use AURKA as a biomarker to predict the chemosensitivity of colon cancer to platinum in the clinic.

## Data Availability

All the data used to support the findings of this study are included within the article.

## Supplementary Materials

Supplementary materials Supplementary Figure 1. The correlation of AURKA level and OS in patients with wildtype TP53 or mutant TP53. A. The patients with the higher AURKA had a longer OS time in TP53 wildtype groups, although only a marginal significance was achieved due to the reduced number of samples. B. No difference was found in TP53 mutant groups. C. Most of the mutations in TP53 were missense variant, followed by stop gained and frameshift variant. Supplementary Table 1. We compared the expression level of AURKA mRNA by calculating the mean value and standard deviation. The effect of AURKA copy number variant (CNV) on AURKA expression level was also analyzed based on the Colon adenocarcinoma (COAD) data from the TCGA database. Supplementary Table 2. The primers for PCR.Supplementary Table 3. The assessment of transcriptional regulatory potential of p53 on DDR genes based on Chip-Seq data from cell lines. Supplementary Table 4. The assessment of transcriptional regulatory potential of p53 on Mismatch Repair (MMR) genes based on Chip-Seq data from cell lines.
