# Supplementary material for: AURKA Increase the Chemosensitivity of Colon Cancer Cells to Oxaliplatin by Inhibiting the TP53-Mediated DNA Damage Response Genes
Source: Biomed Res Int. 2020 Aug 10;2020:8916729. doi: 10.1155/2020/8916729 (PMC7439175; doi:10.1155/2020/8916729)
Supplement: Supplementary materials — Supplementary Figure 1. The correlation of AURKA level and OS in patients with wildtype TP53 or mutant TP53. A. The patients with the higher AURKA had a longer OS time in TP53 wildtype groups, although only a marginal significance was achieved due to the reduced number of samples. B. No difference was found in TP53 mutant groups. C. Most of the mutations in TP53 were missense variant, followed by stop gained and frameshift variant. Supplementary Table 1. We compared the expression level of AURKA mRNA by calculating the mean value and standard deviation. The effect of AURKA copy number variant (CNV) on AURKA expression level was also analyzed based on the Colon adenocarcinoma (COAD) data from the TCGA database. Supplementary Table 2. The primers for PCR.Supplementary Table 3. The assessment of transcriptional regulatory potential of p53 on DDR genes based on Chip-Seq data from cell lines. Supplementary Table 4. The assessment of transcriptional regulatory potential of p53 on Mismatch Repair (MMR) genes based on Chip-Seq data from cell lines. [file 8916729.f1.zip › 8916729.f2.docx]

Supplementary Material

1. Supplementary Figure 1


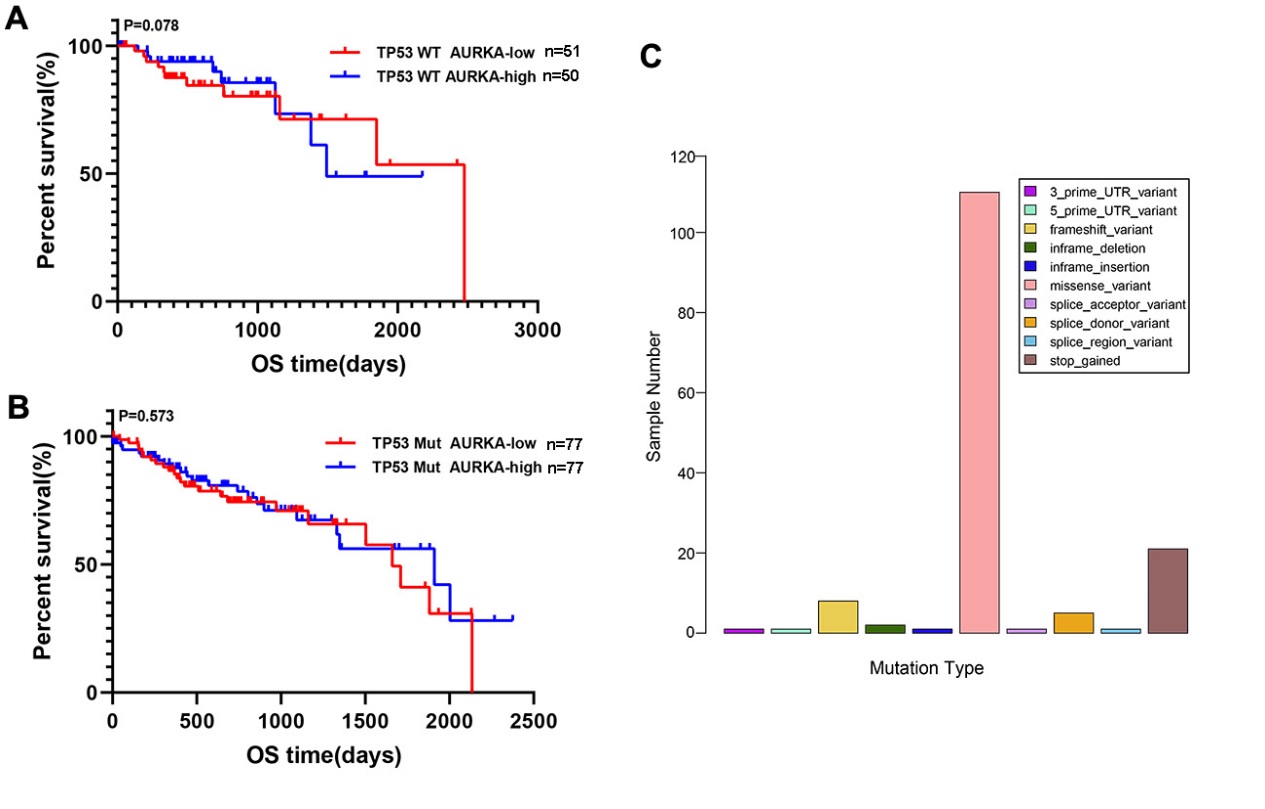


Supplementary Figure 1. The correlation of AURKA level and OS in patients with wildtype TP53 or mutant TP53. A. The patients with the higher AURKA had a longer OS time in TP53 wildtype groups, although only a marginal significance was achieved due to the reduced number of samples. B. No difference was found in TP53 mutant groups. C. Most of the mutations in TP53 were missense variant, followed by stop gained and frameshift variant.

# Supplementary Table 2. The primers for PCR.

| **Genes** | **Primers (5’-3’)** |
| --- | --- |
| ATR | F: TCCGTGATGTTGCTTGAT  R: ATGACAGGAGGGAGTTGC |
| BRCA2 | F: ATAAACAAGCAACCCAAGT  R: TCAAGGAGATGTCCGATT |
| RPA1 | F: GTGGACCATTTGTGCTCGTG  R: TTCGTCAACCAGTTCTAGGGA |
| XRCC1 | F: CCTTTGGCTTGAGTTTTGTACG  R: CCTCCTTCACACGGAACTGG |
| RAD51 | F: AGTGATGTCCTGGATAATGT  R: GACCCGAGTAGTCTGTTCT |
| NHEJ1 | F: GGCCAAGGTTTTTATCACCAAGC  R: TGGGCGAAGGAGATTATCCAAAT |
| GAPDH | F: GGCCTCCAAGGAGTAAGACC  R: AGGGGTCTACATGGCAACTG |

1. **Supplementary Table 3. The assessment of transcriptional regulatory potential of p53 on DDR genes based on Chip-Seq data from cell lines.**

| **Genes** | **TP53 targeting potency** | |  |
| --- | --- | --- | --- |
|  | **MAD-MB-231**  **[PMID: 28411283]** | **Saos-2**  **[PMID: 21459846]** | |
| ATM | 0.953 | 0.325 | |
| ATR | 1.544 | 1.332 | |
| ABL1 | 1.075 | 0.551 | |
| ABL2 | 1.269 | 0 | |
| BRCA1 | 0.719 | 0 | |
| TP53 | 1.705 | 0.248 | |
| CHEK2 (Chk2) | 1.567 | 0.584 | |
| CHEK1 (Chk1) | 0.822 | 0.854 | |
| CDC25A | 1.581 | 0.15 | |
| CDC25B | 2.22 | 0.737 | |
| CDC25C | 2.314 | 0.452 | |
| XRCC6 (Ku70) | 1.876 | 0.561 | |
| XRCC5 (Ku80) | 1.353 | 0.951 | |
| PRKDC (DNA-PK) | 1.099 | 0.734 | |
| DCLRE1C (Artemis) | 1.96 | 0.604 | |
| XRCC4 | 0.942 | 0.921 | |
| LIG4 (DNA ligase IV) | 0.958 | 0.454 | |
| NHEJ1 (XLF) | 2.821 | 1.268 | |
| PARP-1 | 0.448 | 0.121 | |
| XRCC1 | 2.625 | 0.727 | |
| LIG3 (DNA ligase III) | 1.907 | 0.306 | |
| RPA1 (RPA) | 1.001 | 0.731 | |
| PALB2 | 1.417 | 0.079 | |
| BRCA2 | 1.249 | 0.532 | |
| RAD51 | 1.171 | 0.734 | |

All the DDR genes involved in this manuscript were written in red.

| PMID | 30107566 | 25883152 | 24078252 | 25790137 | 28963538 | 28411283 | 24823795 | 21459846 |
| --- | --- | --- | --- | --- | --- | --- | --- | --- |
| Cell Line | 116 | GM00011 | H9 | IMR90 | SW480 | MDA-MB-231 |  | Saos-2 |
| Cell Type | Lymphocyte | Fibroblast | Embryonic Stem Cell | Fibroblast |  |  | Keratinocyte | Osteosarcoma cell |
| ID：Cistrome DB | 90650 | 52167 | 43999 | 52092 | 81528 | 75042 | 44763 | 6548 |
|  |  |  |  |  |  |  |  |  |
| MLH1 Score | 0.224 | 1.162 | 1.198 | 0.96 | 1.212 | 2.288 | 0.015 | 1.281 |
| MSH2 Score | 0.996 | 0 | 1.083 | 0.982 | 1.536 | 1.844 | 0.001 | 0.016 |
| MSH6 Score | 0.121 | 0.104 | 0.106 | 1.375 | 1.408 | 1.897 | 0.104 | 0.551 |
| PMS2 Score | 0 | 0 | 0.993 | 2.054 | 1.817 | 1.042 | 0 | 1.667 |
| PMS1 Score | 0 | 0.001 | 1.215 | 1.22 | 1.297 | 1.689 | 0 | 0.467 |

1. **Supplementary Table 4. The assessment of transcriptional regulatory potential of p53 on Mismatch Repair (MMR) genes based on** **Chip-Seq data from cell lines.**
